# Dover Micro Open Street Events: Evaluation Results and Implications for Community-Based Physical Activity Programming

**DOI:** 10.3389/fpubh.2019.00356

**Published:** 2019-11-22

**Authors:** Richard R. Suminski, Chanda Jackson-Short, Noel Duckworth, Eric Plautz, Karen Speakman, Rita Landgraf, Freda Patterson

**Affiliations:** ^1^Department of Behavioral Health and Nutrition, University of Delaware, Newark, DE, United States; ^2^National Council on Agricultural Life and Labor Research Fund, Inc., Dover, DE, United States; ^3^College of Health Sciences, University of Delaware, Newark, DE, United States

**Keywords:** physical activity, Open Streets, community based participatory action research, built environment, community capacity

## Abstract

Open Streets events provide opportunities for residents to be active. The current program developed and implemented five smaller scale, Micro Open Streets Events (MOSE) in Dover, DE that provided a range of opportunities for physical activity over a <0.5 miles stretch of closed road. Our objective was to evaluate the capacity of this approach to reach residents and improve knowledge and intention to engage in physical activity once the event was over. We used individual surveys, observational, and neighborhood audit factors to assess MOSE participation and conduciveness to physical activity. Our results showed that MOSE attendance ranged from 40 to 500 adults from a high-risk demographic (i.e., non-Caucasian, middle-age, overweight), who demonstrated a strong liking of the MOSE and increased knowledge of, and intention to engage in physical activity following the event. Our data suggest that where a full-scale Open Streets event is not feasible, a MOSE may be a viable alternative.

## Introduction

Consistent with national rates (1), up to half of adults in the state of Delaware are inactive and fail to meet physical activity guidelines (2). Creating or enhancing knowledge about, and access to, safe places for physical activity is part of the national agenda to address insufficient activity (1). Open Streets events that temporarily close street sections to motorized traffic, and provide attendees access to various types of activity opportunities (3), have emerged in response to this national agenda. Evidence suggests that return Open Streets attendees are more active, and that while at the event, the majority engage in 30–150 min of activity (4).

Although there is much evidence to support the positive effects of Open Streets events (5), it is noteworthy that the majority of events are held in large urban centers, and involve the closing of tens of miles of highway (e.g., 4). Moreover, the sustainability of physical activity engagement following the events is not clear. Given evidence to show the importance of social and environmental level factors with physical activity (6, 7), assessment of such factors (i.e., social connectedness, walkability) might be important to informing approaches to sustaining physical activity engagement after the Open Streets event is over.

To expand on the evidence-base in support of the Open Street initiatives, a local multi-interest organizing committee was formed to develop, implement, and evaluate five “Micro” Open Streets events between June and December 2018 in the small city of Dover (37,109 residents) (US Census). These events were considered “Micro” because unlike other published accounts of Open Streets events that involved closing ~70 miles of highways for recreation (8), the current “Micro” events closed 2–4 blocks (i.e., 0.2–0.4 miles). In this report, we detail our Micro Open Street Event (MOSE) program objectives, intervention components, evaluation plan and results, as well as the implications of our findings for the public health field.

## Purpose and Objectives

The purpose of our MOSE program was to provide healthy lifestyle opportunities to the residents of Dover, DE and to increase their knowledge about, and intention to, participate in healthy lifestyle activities (e.g., physical activity) beyond attending the MOSE. The objective of the current study was to conduct a multi-level evaluation of the MOSE in order to describe the following:

(1) Individual-level characteristics: Attendee demographics, perceptions of the MOSE, physical activity behaviors, perceived neighborhood support for physical activity (e.g., safety, facilities), social connectedness, and health status;
(2) Program-level characteristics: Estimates of event attendance and participation at different event activities (e.g., jump rope);
(3) Neighborhood-level characteristics: Quantify the walkability, conditions, aesthetics, safety, and design of the area surrounding the MOSEs.

## Intervention Approach

### Overview of Dover MOSEs

The MOSEs were conceptualized by a local “Healthy Neighborhood” council developed out of the state healthcare transformation plan to bring together healthcare systems and multi-sector community partners to improve population health and priority health areas. The MOSEs as an intervention aligned with a Comprehensive Neighborhood Revitalization Plan led by National Council on Agricultural Life and Labor Research Fund, Inc. (NCALL) and were implemented by a multi-interest organizing committee that included representatives from the city's Parks and Recreation staff, local Universities, the city's Children's Theater, community volunteers, a local college drug and alcohol prevention program, a local health system. The organizing committee was chaired by staff from NCALL through the Restoring Central Dover program. NCALL served as a fiscal agent for the state innovation model funding, which provided the funding for the MOSEs. Funding was also provided by Restoring Central Dover's Community Engagement work group. The MOSE's concept fit naturally with the work that was being accomplished under the Restoring Central Dover endeavor and was an expansion of Play Streets sponsored by the Community Engagement work group. The committee had standing monthly meetings beginning 2 months prior to the first MOSE, and met bimonthly and as-needed thereafter to guide further event offerings. It is important to note that NCALL received the SIM funding in June 2018 and funds had to be spent by January 2019.

### Event Timing and Locations

One MOSE was held monthly between June and November, 2018 (*N* = 5); the July event was canceled due to inclement weather and rescheduled to November. Each event was planned around, and anchored to, an existing community event. For example, the August MOSE occurred in conjunction with a back-to-school event where school supplies were distributed. The MOSE dates/times and, where applicable, concurrent community events were as follows: Event 1, Saturday June 30, 10 a.m.−2 p.m.: Agricultural Day (Dover business district); Event 2, Wednesday August 22, 5–7 p.m.: School supply giveaway (Dover low-income, minority neighborhood); Event 3, Sunday, September 16, 1–4 p.m.: no concurrent community event, but was an effort to involve and engage the faith community (Dover low-income, minority neighborhood); Event 4, Saturday, October 20, 8–11 a.m.: Mayors' Events Challenge, 5-K run, kayak race (Dover urban park); and, Event 5, Thursday, November 29, 5–7: City of Dover's Capital Holiday Celebration located in the Small Business District.

### Event Activities

Each MOSE offered activities that were intended to provide attendees with opportunities to engage in, and learn about, physical activity and healthy eating. To the greatest extent possible, activities were administered by community-based organizations (for a more extensive list of participating organizations see Appendix A) so that attendees could have the chance to engage with groups that were already in their communities. Examples of activities include a basketball and football station administered by the Police Athletic League, a bike rodeo, healthy eating demonstrations and mock grocery shopping challenges, and a stage-based station that had other organized physical activity classes such as Zumba, jazzercise, yoga, soul line dancing, cardio-drumming, a boot camp style workout and more.

## Evaluation Methods

A multi-level evaluation design that assessed individual, program, and neighborhood factors related to the MOSEs and the attendees was implemented. Five data collectors completed a 4–6 h training session on data collection procedures prior to the first MOSE. The manual-based training comprised of a didactic review of Open Streets events, a review of data collection tools, and practice simulations of the data collection procedures.

### Individual-Level Measures/Tools Used

Consistent with previous Open Streets events (9), data collectors positioned at the 2–3 entry/exit points, asked every third adult leaving the event if they would like to complete an anonymous survey. Willing attendees were asked their age and if they spoke and understood English. Attendees meeting these eligibility criteria were given the 33-item survey to complete. If the attendee completing the survey was in a group, the other group members were also invited to participate. Data collectors answered any participant questions, checked surveys for completeness, and collected surveys following completion.

The demographic factors of self-reported age, birth sex (male/female), race (White, non-Hispanic, Black or African American, American Indian or Alaska Native, Pacific Islander, Latino, Multiracial, other), self-reported height and weight [to calculate body mass index (BMI)], and social media use were evaluated. Social media use was assessed using a single item that asked participants to check which social media outlets they used; options were Facebook, Twitter, Instagram, Snapchat, YouTube, LinkedIn, Pinterest, Tumblr, Other. Liking of event and intention to attend future a MOSE were also assessed using a 5-point Likert scale (0 = strongly disliked−4 = strongly liked); past MOSE attendance was assessed using a single item soliciting a yes/no response. Current physical activity levels were measured using a 7-day recall survey that asked attendees to report the number of days active, and minutes active on each active day, for the last week. Intentions to participate in physical activity and knowledge about, and use of local recreational facilities because of attending MOSE were also assessed using multiple point and Yes/No responses. Social connectedness was measured using questions adapted from Williams (10). Referring to a 5-point Likert scale (0 = strongly disliked−4 = strongly liked), attendees indicated the degree to which there are people in their community they can trust and turn to for advice and whether interacting with people in their community influences their interest in “things that happen” and “new things.” Attendees also rated their general health on a 5-point Likert scale and reported the number of days during the last month their health was poor.

### Program-Level Measures/Tools Used

At the program level, MOSE check-in sheets were used to estimate event attendance. Event activities and participation were assessed using Ecological Momentary Assessment (EMA). At the start of each MOSE, a data collector surveyed and recorded each activity and categorized according to “type.” Event types could be classified as: physical activity opportunity; food/diet opportunity; physical activity education/information; food/diet education/information. During the last 15 min of each hour, the data collector walked the length of the event and recorded the number of attendees at each activity. The EMA method provides an estimate of event participation.

### Neighborhood-Level Measures/Tools Used

During the hour prior to each MOSE, a data collector walked all routes (sidewalks and streets) within a 0.25 radius of the MOSE to assess neighborhood-level characteristics associated with walking (11). A standard audit form was used to obtain information on the presence/absence of certain environmental factors representing conditions (e.g., sidewalks), safety (e.g., crosswalks), facilities (e.g., community facilities), and aesthetics (e.g., trees).

## Data Management and Analysis

Data from completed source forms were entered into SPSS and quality assurance checks were made on a 10% random selection of data points. Descriptive statistics consisting of Means (M) ± Standard Deviations (SD) for continuous variables, and percentages for categorical variables were generated. Data management and analyses were performed using the SPSS statistical software package (IBM Corp. 2015. SPSS Statistics for Windows, Version 23.0. Armonk, NY: IBM Corp.).

## Results

### Individual-Level Characteristics (Objective 1)

#### Attendee Demographics

Seventy-eight attendees completed a survey (64.9% response rates). As shown in Table 1, attendees were mostly female (68%), middle-aged (Mean age = 41.5 years; *SD* = 13.2), and African American (58%). A wide array of websites/applications were used with Facebook (83%), Instagram (33%), and YouTube (37%) being the top programs. In terms of health status, mean BMI was 28.4 (*SD* = 6.3), and overall, 32.8% were overweight (BMI 25–29) and 34.5% were obese (BMI ≥ 30). Approximately one in four (23%) respondents stated their health was “fair” or “poor;” respondents reported poor mental or physical health on 2.7–4.3 days in the last month.

**Table 1:** Sample characteristics of MOSE attendees.

**Total attendees surveyed**	**78**
**Sex [*****N*** **(%)]**	
Male	24 (31.2)
Female	53 (68.8)
Age [M;SD]	41.9;12.2
**Race [*****N*** **(%)]**	
White, non-Hispanic	21 (28)
Black/African American	45 (60)
American Indian/Alaska Native	0
Latino	3 (4)
Multiracial	3 (4)
Other	3 (4)
**Social media outlet use [*****N*** **(%)]**	
Facebook	65 (83.3)
Twitter	12 (15.4)
Instagram	24 (30.8)
Snapchat	10 (12.8)
YouTube	29 (37.2)
LinkedIn	9 (11.5)
Pinterest	10 (12.8)
Tumblr	1 (1.3)
Body Mass Index (kg/m^2^; M;SD)	28.4;6.3
**Self-rated general health [*****N*** **(%)]**	
Excellent	22 (31)
Very good	33 (46)
Fair	17 (22)
Poor	0
Days last month physical health not good [M;SD]	3.7;7.7
Days last month mental health not good [M;SD]	4.3;8.1
Days last month poor health hindered activities [M;SD]	2.7;5.8

#### MOSE Experiences, Attendance, and Participation

Almost all attendees surveyed strongly liked the MOSE (82%), 41% attended a previous Open Street event/MOSE, and 80% planned to attend in the future. While at the MOSEs, walking was the most common physical activity reported (*M* = 30.8 min, *SD* = 36.9) (Table 2).

**Table 2:** MOSE liking, participation, and intention to attend future Open Streets Events.

**Liking of MOSE [*****N*** **(%)]**	
Strongly liked	59 (80.8)
Somewhat liked	11 (15.1)
Neither liked or disliked	3 (4.1)
Somewhat disliked	0
Strongly disliked	0
Attended a previous Open Streets Event [*N* (%)]	29 (41.4)
**Likely to attend a future Open Streets Event [*****N*** **(%)]**	
Very likely	58 (80.6)
Likely	12 (16.7)
Neither likely/unlikely	2 (2.8)
Unlikely	0
Very unlikely	0
**Activities participated in at MOSEs [*****N*** **(%)]**	
Walking	55 (70.5)
Bicycling	4 (5.1)
Shopping	16 (20.5)
Visited restaurant or pub	4 (5.1)
Other activities	10 (12.8)
**Time spent at activities [M;SD]**	
Walking	26.3;35.7
Bicycling	1.9;9.2
Shopping	4.3;16.3
Visited restaurant or pub	0.59;3.8

#### Physical Activity and Environmental Support

Walking outside was the most common form of physical activity participated in during the previous 7 days (62%). Few attendees said they cycled or ran/jogged outside during this time period (12% for both). Nearly all respondents intended to be physically active in the next 7 days (88%), and 43% said this was because of attending the MOSE. In terms of neighborhood factors related to being physically active, 55% indicated having local recreation facilities, 49% learned of new local recreation facilities because of attending a MOSE, 64% rated their local recreation facilities as very good to excellent, 48% reported using these facilities often/very often, and 48% stated their use of such facilities would be higher because of attending the MOSE. Most (91%) attendees reported feeling safe in their neighborhood (Table 3).

**Table 3:** Physical activity behavior and environmental factors related to physical activity behavior.

**Current physical activity participation [*****N*** **(%)]**	
Walked outside in last 7 days	47 (61.8)
Biked outside in last 7 days	9 (11.8)
Ran/Jogged outside in last 7 days	9 (11.8)
**Days during past week spent in Specific Physical Activities (M;SD days/weeks)**	
Walking outside	2.7;2.6
Biking outside	0.3;1.1
Running/Jogging outside	0.2;0.6
**Intend on being physically active in the next 7-days [*****N*****, %]**	
Strongly agree	52 (68.4)
Somewhat agree	15 (19.7)
Neither agree/disagree	5 (6.6)
Somewhat disagree	1 (1.3)
Strongly disagree	3 (3.9)
**More likely to be physically active in the next 7-days because of attending a MOSE [*****N*****, %]**	
Strongly agree	33 (42.9)
Somewhat agree	18 (23.4)
Neither agree/disagree	21 (27.3)
Somewhat disagree	2 (2.6)
Strongly disagree	3 (3.9)
Have local recreational facilities [*N*, %]	56 (73.7)
Learned about local recreational facilities at this MOSE [*N*, %]	37 (49.3)
Perceived quality of recreational facilities in local area [*N*, %]	
Excellent	19 (27.5)
Very good	26 (37.7)
Fair	20 (29.0)
Poor	4 (5.8)
**How often recreational facilities in local area used [*****N*****, %]**	
Very often	17 (23.6)
Often	17 (23.6)
Sometimes	25 (34.7)
Never	13 (18.1)
**Extent to which Open Streets events will increase use of local recreational facilities [*****N*****, %]**	
Very much	34 (47.9)
Somewhat	31 (43.7)
Not at all	6 (8.5)
**Frequency of feeling safe in the Neighborhood [*****N*** **(%)]**	
Always	30 (41.1)
Most of the time	36 (49.3)
Sometimes	7 (9.6)
Rarely	0
Never	0

#### Community Connectedness

Overall, the sample reported a strong sense of community connectedness with 60% having several people in their community they trusted to help solve their problems. Most (80–84%) said that interacting with community members increased their interest in the community, and that such interactions made them want to try new things. Overall, the attendees reported moderate (*M* = 11.6; *SD* = 3.7) levels of connectedness with their community (Table 4).

**Table 4:** Community connectedness reported by MOSE attendees.

**“I have someone to trust” [*****N*****, %]**	
Strongly agree	19 (26.0)
Somewhat agree	25 (34.2)
Neither agree/disagree	18 (24.7)
Somewhat disagree	6 (8.2)
Strongly disagree	5 (6.8)
**“I have someone to turn to for advice” [*****N*****, %]**	
Strongly agree	20 (27.4)
Somewhat agree	26 (35.6)
Neither agree/disagree	17 (23.3)
Somewhat disagree	4 (5.5)
Strongly disagree	6 (8.2)
**Interacting with community members increases interest in town [*****N*****, %]**	
Strongly agree	32 (43.8)
Somewhat agree	26 (35.6)
Neither agree/disagree	8 (11.0)
Somewhat disagree	3 (4.1)
Strongly disagree	4 (5.5)
**Interacting with community members makes me want to try new things [*****N*****, %]**	
Strongly agree	33 (45.2)
Somewhat agree	28 (38.4)
Neither agree/disagree	6 (8.2)
Somewhat disagree	2 (2.7)
Strongly disagree	4 (5.5)
Total community connectedness scale [0–16; M;SD]	11.6;3.7

### Program Level Characteristics (Objective 2)

Data from the event logs showed that between 50 and 500 adult attendees registered for each MOSE. Event two, that occurred in August and coincided with the distribution of back to school supplies, was the most widely attended (*N* = 500). There were a total of 18 activities that were categorized in one of the following classifications: physical activity opportunity, food/diet opportunity, food/diet education, physical activity education, and other (Table 5). The physical activity opportunity category was most commonly represented (66% of all activities) with 12 different stations and included activities such as jump rope and bouncy castle. Three activities were observed in each of the food/diet opportunity (i.e., fresh market food stand) and food/diet education (i.e., Food Bank of Delaware) categories. No activities explicitly addressed physical activity education, and seven “other” activities were present (e.g., face painting, the local public library, smoking cessation).

**Table 5:** MOSE activity classifications and number of attendees at each activity.

**Activity classification**	**Activity**	^*^# Attendees observed at MOSE activities			
		**1st MOSE**	**2nd MOSE**	**3rd MOSE**	**4th MOSE**
Physical activity opportunity	Bike rodeo	0	**X**	0	**X**
	Fun stations (e.g., bean bag toss, net)	5	51	10	**X**
	Stage (Zumba, jazzercise, boot camp)	16	**X**	13	**X**
	Jump rope	9	**X**	**X**	**X**
	Police—youth football/basketball	25	**X**	**X**	**X**
	National Guard—football throw	**X**	22	**X**	**X**
	Dover parks and recreation	**X**	24	**X**	**X**
	Delaware Department of Transportation	4	29	**X**	**X**
	Bounce castle	3	**X**	15	**X**
	Laser tag	**X**	**X**	20	**X**
	Kayaking	**X**	**X**	**X**	20
	5 K walk/run	**X**	**X**	**X**	30
Food/diet opportunity	Fresh market food stand	7	**X**	**X**	**X**
	Food truck (free)	8	29	**X**	**X**
	Food station (free)	23	92	10	**X**
Food/diet education	Your Community Garden	**X**	0	**X**	**X**
	Food Bank of Delaware	**X**	10	**X**	**X**
	University Agricultural Education display	**X**	**X**	14	**X**
Physical activity education	None offered				
Other	Delaware Quit Line (smoking cessation)	6	**X**	**X**	**X**
	Dover Public Library	**X**	14	**X**	**X**
	Buffalo Soldiers Motor Club	**X**	16	**X**	**X**
	Capital School District	**X**	16	**X**	**X**
	Delaware Public Health and Social Services	**X**	23	**X**	**X**
	Face painting	**X**	**36**	**X**	**X**
	Church community center table	**X**	**X**	**X**	**X**

* *Event 5 activities were not observed; X, the activity was not offered at this MOSE*.

Most stations offering physical activity opportunities were well-attended. Placement and weather may have impacted activity participation. The bike rodeo was underutilized and this was apparently due to its location off to the side or at the non-entrance/exit end of the event. The bounce house and fun stations were underutilized at event 1, most likely due to the extreme heat (96°F) on the day of that event.

### Neighborhood-Level Characteristics (Objective 3)

The routes surrounding the MOSEs were local (i.e., residential) with on-street parking and sidewalks on both sides (Table 6). Measures to enhance pedestrian safety were lacking regarding the presence of speed limit signs, crossing signals, and curb extensions (which allow pedestrians better views of traffic at street corners); however, pedestrians were separated from the road (by a grassy median) and easy to see. Although bike paths were mostly absent, the areas displayed a mix of residential and commercial/retail space as well as community facilities (e.g., tennis courts). The areas around the routes were generally aesthetically pleasing having trees and attractive buildings. The overall impression was that the routes were inviting for all in terms of walkability, except for the routes associated with event 2. The routes surrounding this event were less safe and aesthetically pleasing and they had fewer facilities than the routes near events 1 and 3.

**Table 6:** Walkability of routes surrounding open streets events.

**MOSE (#routes audited)**	**1 (*n* = 5)**	**2 (*n* = 10)**	**3 (*n* = 6)**
**Environmental aspect**	**Percentages of routes displaying aspect**		
**Street description**			
Street type—local	40	100	83
2 lanes	100	100	100
Parking both sides	80	100	100
Sidewalk both sides	100	100	100
**Safety**			
Car speed 30 mph	60	10	17
Speed limit signs	0	0	0
Pedestrians separated from road	80	90	100
Crossing signals	0	0	33
Crosswalks	80	10	33
Pedestrians easy to see	100	80	100
Curb extensions	20	0	0
Street lights	100	89	100
**Facilities**			
Mixed land use	80	40	67
Community facilities	80	20	67
Bike paths	40	0	17
**Aesthetics**			
Attractiveness of sidewalks			
Poor	0	20	17
Medium	20	60	33
Good	80	20	50
Trees	100	40	100
Attractive buildings	100	0	17
Trash cans	60	0	17
**Overall impression**			
Route is inviting for all	100	40	83

## Implications for Public Health

Through the current program, we assessed the attendance and attendee characteristics of five MOSEs in Dover, Delaware. We also examined the knowledge about physical activity opportunities, and intention to engage in these activities because of attending a MOSE. Given that Open Streets events in the past have utilized up to 70 miles of closed road-way (8), our MOSE that used between 0.2 and 0.4 miles of roadway, is a novel, arguably more easily disseminated and feasible model. The current evaluation adds to this literature by demonstrating the reach and potential impact of these smaller scale events, while for the first time, considering the neighborhood walkability and aesthetics that are known to impact long term physical activity participation (12).

Some of the main findings from this evaluation were the capacity to deliver a range of activities that reached up to 500 local residents, who could be characterized as a priority population. Specifically, attendees were middle-aged, non-Caucasian, overweight, and mostly inactive. Moreover, almost one quarter reported that their health was fair or poor. These characteristics are demonstrated risk factors for poor health outcomes (13, 14) and thus emphasize the capacity for these MOSEs to reach at-risk populations.

Another key finding of this evaluation was the high degree of “liking” espoused by 82% of survey respondents, and the strong likelihood of attending another. Importantly, respondents reported being active for ~36 min while at the event, and that attending the event increased their knowledge about venues for activity in their community and intention to be more active. While sustaining a regular physical activity habit is the long-term goal for any physical activity program, identifying “windows of opportunity” through which high-risk individuals can be “exposed” to healthy lifestyle options and encouraged to initiate regular healthy habits, is an important community health initiative. These data suggest that the MOSEs described in this study may be such a conduit, especially for physical activity.

Critical to sustaining physical activity are having social and environmental supports (15). The current evaluation showed that about half of the respondents felt they had someone to trust, and that their local recreational facilities were of good quality while the majority of respondents felt safe in their neighborhood. Importantly, the neighborhood audits showed a preponderance of features shown to be positively associated with regular physical activity including high levels of perceived neighborhood safety (16), aesthetically pleasing surroundings, community facilities, and generally inviting routes (7). It may be important to consider such factors when selecting locations for MOSEs. For example, if neighborhood walkability and aesthetics are low, it may be important to provide event activities that exhibit physical activity opportunities that do not rely on being outside in the neighborhood. Likewise, the environmental conditions surrounding a MOSE or any Open Streets event, have the potential to impact event attendance (positively or negatively), and thus warrant consideration in the evaluation strategy.

This cross-sectional study relies on self-reported data from a sub-set of MOSE attendees. A comparison between attendees who completed the survey vs. those that did not was not possible, thus there is the possibility that our findings are influenced by a response bias. Future evaluations on MOSEs should examine participation in healthy lifestyle behaviors such as physical activity following the event, and conduct a multivariable examination of demographic, health status, and neighborhood factors that may affect the likelihood of engaging in such behaviors following a MOSE. Also important would be to consider how to further build on the community connections between MOSE activities and established community resources for healthy lifestyle behaviors so that there can be greater continuity from the single (or even repeated) MOSE, with the established community resources.

In conclusion, this study provides a positive signal for a MOSE as an alternative when a full-scale Open Streets event is not feasible. Continued efforts in this area, as described above, have the potential to advance community-based approaches to encouraging high-risk populations to engage in healthy lifestyle behaviors.

## Summary Box

**What is already known on this topic?**

Insufficient physical activity remains a public health priority. Open streets events provide opportunities for residents to be active.

**What is added by this report?**

Micro Open Streets Events that cover <0.5 miles are effective in delivering a range of healthy lifestyle behaviors to at-risk populations. Attendees to the MOSEs engage in physical activity, learned about community venues to be active, and reported high levels of intentions to be physically active due to attending the MOSE.

**What are the implications for public health practice?**

Where a full-scale Open Streets event is not feasible, a MOSE may be an efficacious alternative.

## Data Availability Statement

The datasets generated for this study are available on request to the corresponding author.

## Ethics Statement

The program was approved by the University of Delaware Institutional Review Board (#1239506). Given the observational nature of the study, and that non-identifiable information was collected as part of the evaluation, participant informed consent was not collected.

## Author Contributions

RS and EP led data collection. FP and RS conducted the analysis, drafted, and completed the final version of the manuscript. RS, EP, FP, CJ-S, ND, KS, and RL conceptualized the program, reviewed, revised, and approved the manuscript.

### Conflict of Interest

The authors declare that the research was conducted in the absence of any commercial or financial relationships that could be construed as a potential conflict of interest.
