# Supplementary material for: Dover Micro Open Street Events: Evaluation Results and Implications for Community-Based Physical Activity Programming
Source: Front Public Health. 2019 Nov 22;7:356. doi: 10.3389/fpubh.2019.00356 (PMC6883340; doi:10.3389/fpubh.2019.00356)
Supplement: Supplementary file 1 [file Table_1.DOCX]

**Appendix A: Other Participating Organizations**

- Dover Parks and Recreation
- The Children’s Theater
- Bayhealth Medical Center
- Delaware Food Bank
- Dover Library
- Wesley College Drug and Alcohol Prevention Program
- University of Delaware- Family and Consumer Science Program Cooperative Extension, Associate of Arts Program, College of Agriculture and Natural Science, Partnership for Healthy Communities
- Delaware State University 4-H and Youth Development Program, and the Expanded Food Nutrition and Education Program
- Greek Organizations
- Wesley United Methodist Church
- John Wesley AME Church
- Calvary Baptist Church
- Solid Rock Baptist Church
- Mt. Zion AME Church
- Maranatha Life Changing Church
- YMCA
- National Guard
- Dover Police Department and the Police Athletic League
- Club Fitness
- DelDOT
- Delaware Tourism
- Herbal Life
- Delaware Quit line
- La Red
- AmeriHealth Caritas Delaware
- Buffalo Soldiers
- Delaware Public Health and Social Sciences
- Dover Tents and Events
- Communities in Schools
- Delaware Parents Association
- Capital School District
- First State Dance Academy
- C&K Soul Line
